# Standardized Comparison of Cardiovascular Risk Factors Prevalence in Spanish Women and Men Living with HIV and in the General Population

**DOI:** 10.3390/jpm11111085

**Published:** 2021-10-25

**Authors:** Anna Camps-Vilaró, Silvia Pérez-Fernández, Isaac Subirana, Ramon Teira, Vicente Estrada, Pere Domingo, Irene R. Dégano, Jaume Marrugat

**Affiliations:** 1 REGICOR Study Group, IMIM (Hospital del Mar Medical Research Institute), Barcelona Biomedical Research Park (PRBB), Dr. Aiguader 88, 08003 Barcelona, Spain; acamps@imim.es (A.C.-V.); silvia_medina2712@hotmail.com (S.P.-F.); isubirana@imim.es (I.S.); 2 CIBER of Cardiovascular Diseases (CIBERCV), Instituto de Salud Carlos III (ISCIII), 28029 Madrid, Spain; 3 Biocruces Bizkaia Health Research Institute, 48903 Barakaldo, Spain; 4 Infectious Diseases Unit, Hospital Sierrallana, 39300 Torrelavega, Spain; ramon.teira1@gmail.com; 5 Infectious Diseases Unit, Hospital Clínico San Carlos, 28040 Madrid, Spain; vesda001@gmail.com; 6 Department of Infectious Diseases, Hospital de la Santa Creu i Sant Pau, Institut de Recerca del Hospital de la Santa Creu i Sant Pau, 08041 Barcelona, Spain; pdomingo@santpau.cat; 7 Faculty of Medicine, University of Vic-Central University of Catalonia (UVic-UCC), 08500 Vic, Spain

**Keywords:** HIV, people living with HIV, prevalence, cardiovascular risk factor, general population

## Abstract

People living with HIV (PLWH) have an increased risk of cardiovascular (CV) disease, likely due to a higher prevalence of CV risk factors. We compared the age-standardized prevalence and management of CV risk factors in PLWH to that of the general population in Spain. Blood pressure, lipid, glucose, and anthropometric profiles were cross-sectionally compared along with the treatment of hypertension, dyslipidemia, and diabetes in a general population cohort and a PLWH cohort. Prevalence rates were standardized by the direct method by 10-year age groups in European standard populations and stratified by gender. We included 47,593 individuals aged 35 to 74 years, 28,360 from the general population cohort and 19,233 from the PLWH cohort. Compared to the general population, PLWH had a higher concentration of triglycerides (>35 mg/dL in women and >26 mg/dL in men) and a higher prevalence of smoking (>23% and >17%) and diabetes (>9.9% and >8.5%). The prevalence of treated diabetes, hypertension, and dyslipidemia were up to three-fold lower in both women and men living with HIV. There was a significant difference in PLWH compared to the general population in the lipid, glucose, and anthropometric profile. In addition, PLWH were less often treated for diagnosed diabetes, hypertension, and dyslipidemia.

## 1. Introduction

The introduction of combined antiretroviral therapy (cART) in the 1990s significantly reduced the morbidity and increased the survival of people living with HIV (PLWH). cART has become the standard of care for PLWH, leading to lifelong suppression of viral replication. cART has steadily improved undetectable viral load at 48 weeks from 48% in 1995 to 78% in 2010 and >90% in the most recent analysis [1]. This success has gained several decades of life expectancy for these patients. As PLWH live longer with their disease, they face age-related chronic comorbidities [2].

PLWH tend to smoke and develop cardiovascular (CV) diseases, hypertension, diabetes, and chronic kidney disease more often than the general population [3,4,5]. PLWH have been reported to have almost 60% greater prevalence of hypertension, coronary artery disease, peripheral arterial disease, or chronic kidney disease as compared to the general population [6]. This increase is probably due to a combined effect: (1) higher prevalence of CV risk factors; (2) chronic inflammation and immune activation from the host response; (3) adverse events of cART; and (4) metabolic effects induced by HIV infection [7,8,9]. These factors may also contribute to the management of PLWH and their comorbidities. Half of PLWH have one or more comorbidities and receive at least one medication concomitant to cART [10].

While comparison of CV risk factors’ prevalence in PLWH and the general population has been reported in some studies, age-standardized risk factor prevalence has never been used to control for differences in age distribution. For instance, PLWH tend to be younger than the general population in these studies. Furthermore, few studies have focused on the sex-related distribution of CV risk factors in PLWH, and available results are biased toward men living with HIV (MLWH). Women living with HIV (WLWH) continue to be under-represented in clinical trials and epidemiological studies, despite representing over half of PLWH worldwide, and their socio-economic and clinical conditions can be different than what is observed in men [11,12]. In Spain, the prevalence of HIV infection is lower among women (0.1%) than in men (0.65%), a distribution that is characteristic of high-income countries and prompts differential analysis by sex [13].

The objective of the present study was to compare the age-standardized prevalence and management of CV risk factors in Spanish WLWH and MLWH with that of the Spanish general population.

## 2. Methods

### 2.1. Study Design and Population

We used a cross-sectional design and two large Spanish cohorts to compare the prevalence of CV risk factors in the general population with that in PLWH aged 35 to 74 years. We excluded individuals outside the defined age range and those records with missing data. For each individual included, we analyzed the most recent measurements available.

Data on the general population were obtained from the epidemiology wing of a study of dyslipidemia, atherosclerotic risk, increased high-sensitivity C-reactive protein (hsCRP), and inflammatory and oxidative status (DARIOS study). DARIOS is a pooled cohort of individual data from 11 population cohorts recruited in 10 Spanish autonomous communities in 2000–2010, which included 28,887 participants aged 35 to 74 years at baseline [14,15].

Data on PLWH were obtained from the VACH cohort, a Spanish registry of PLWH consecutively recruited since 1997 at 23 hospital-based HIV clinics nationwide. The VACH cohort includes 29,217 HIV patients who were 16 years or older at hospital admission or at recruitment to the cohort [16,17].

### 2.2. Data Collection and Measurements

Measurements and questionnaires from the DARIOS and VACH cohorts were obtained during an examination and interview at study inclusion and at their first clinic visit, respectively. Standardized World Health Organization questionnaires [18] were used to collect information on sociodemographic variables, smoking status, and previous history of high blood pressure, dyslipidemia, and diabetes. Physical examinations and blood tests were performed to obtain data on anthropometrics, blood pressure, lipid profile, and glycaemia.

Weight, height, and waist circumference were measured by professional healthcare workers. Body mass index (BMI) was calculated as weight divided by squared height (kg/m^2^). Systolic and diastolic blood pressures were measured in seated individuals with an automatic sphygmomanometer after a 5 min rest. Two measures were taken, and the lowest was recorded. Hypertension was defined as previous diagnosis or treatment and/or systolic blood pressure ≥140 mmHg or diastolic blood pressure ≥90 mmHg.

In DARIOS, blood samples were taken after 10–14 h fasting and stored at −80 °C. Total cholesterol, high-density lipoprotein cholesterol (HDL-c), triglycerides, and glucose were determined using enzymatic methods. Low-density lipoprotein cholesterol (LDL-c) was calculated with the Friedewald equation when triglycerides were <300 mg/dL. Diabetes was defined as (i) previous diagnosis or treatment or (ii) glucose >125 mg/dL. Quotients of total cholesterol/HDL-c and triglycerides/HDL-c were also calculated. VACH measurements were taken from hospital laboratories’ records.

Metabolic syndrome was defined as the presence of three or more of the following five conditions: BMI > 30, waist circumference ≥ 102 cm for men or ≥88 cm for women, triglycerides ≥ 150 mg/dL, HDL-c < 40 mg/dL for men or <50 mg/dL for women, blood pressure ≥ 130/≥85 mmHg, or fasting glucose ≥ 100 mg/dL.

### 2.3. Statistical Analysis

The effect of age differences between the VACH and DARIOS cohorts was controlled by direct standardization of risk factor prevalence using the 1976 European Standard Population (ESP) [19]. The 1976 ESP was chosen over the 2013 ESP to allow direct comparison with previous publications (we used the weights 14, 14, 11, and 7 for the age groups of 35–44, 45–54, 55–64, and 65–74 years, respectively) [20].

Categorical variables including age-standardized prevalences were described as frequencies and compared using the chi-squared test. Continuous variables were described with the mean and 95% confidence interval (CI) and compared using the Student t-test. Triglycerides were log-transformed to normalize their distribution.

All statistical analysis was performed with the R software version 3.6.2 [21].

### 2.4. Ethics

All participants were duly informed and signed their consent to participate. The present study was approved by the Parc de Salut Mar Drug Research Ethics Committee (Authorization CEIm PSMAR 2018/8347-I).

## 3. Results

In this study, we included 47,593 individuals aged 35–74 years, 28,360 from the general population (DARIOS cohort) and 19,233 with HIV (VACH cohort). We excluded 527 individuals from the DARIOS and 9984 from the VACH cohort due to missing data or age out of range (Figure S1).

The proportion of women was 53.5% (15,159) in DARIOS and 23.4% (4,495) in VACH (*p*-value < 0.001). General population individuals were older than PLWH, with a mean age of 54 (95% CI: 53–54) and of 45 (95% CI: 44–45), respectively (*p*-value < 0.001).

Differences in CV risk factor prevalence between women from the general population and WLWH are presented in Table 1. WLWH had significantly higher systolic blood pressure, triglyceride, and glucose concentration levels. WLWH had also higher age-standardized triglycerides/HDL-c quotient and higher prevalence of diabetes, metabolic syndrome, and smoking. WLWH had lower age-standardized concentration of HDL-c, LDL-c, and total cholesterol and lower prevalence of obesity than women from the general population.

On the one hand, the analysis of crude prevalences showed similar results except for the following parameters. Systolic blood pressure, glucose concentration levels, and prevalence of hypertension and metabolic syndrome were lower in WLWH. On the other hand, concentration of triglycerides, HDL-c, LDL-c, and total cholesterol, triglycerides/HDL-c rate, and prevalences of diabetes, obesity, and smoking were similar between WLWH and the general population.

Table 2 presents CV risk factor prevalence in men. Compared to men from the general population, MLWH had a higher concentration of triglycerides, quotient of triglycerides/HDL-c, and prevalence of diabetes, metabolic syndrome, and smoking, but lower age-standardized blood pressure, HDL-c, LDL-c, and total cholesterol concentrations, quotient of total cholesterol/HDL-c, and prevalence of obesity.

When comparing crude and standardized prevalences in MLWH, we found the following differences. Crude estimates for MLWH yielded lower glucose concentration, hypertension, and metabolic syndrome prevalence, and similar concentration of blood pressure, triglycerides, HDL-c, LDL-c, and total cholesterol, quotients of total cholesterol/HDL-c and triglycerides/HDL-c, and prevalence of diabetes, obesity, and smoking than in men from the general population.

The percentage of individuals with treated hypertension, diabetes, and dyslipidemia was lower in PLWH than in the general population in both women and men (Table 1 and Table 2). In WLWH, we estimated a 2.9-fold decrease in the prevalence of treated hypertension, a 2.8-fold decrease in treated diabetes, and a 1.6-fold decrease in treated dyslipidemia compared to their general population counterparts. Similar differences were observed in MLWH (2.2-, 3.0-, and 1.5-fold decreases, respectively) compared to men in the general population when prevalences were age-standardized.

## 4. Discussion

This study compared CV risk factor prevalence between Spanish PLWH (VACH cohort) and the Spanish general population (DARIOS cohort), stratified by sex and, importantly, standardized for age. The most prevalent CV risk factors observed in PLWH were a higher triglycerides concentration (>35 mg/dL higher in women and >26 mg/dL higher in men) and a higher prevalence of smoking (>23% and >17%, respectively) and diabetes (>9.9% and >8.5%) as compared to the Spanish general population.

The higher concentration of triglycerides observed in the VACH cohort agrees with results of the United States study by Önen et al. [22], the Copenhagen study by Gelpi et al. [23], and the French study by Savès et al. [24]. The French cohort was receiving cART with protease inhibitors (PI), which could have increased triglycerides concentration. Most PLWH from the VACH cohort also received cART containing a PI or a nucleoside reverse transcriptase inhibitor (NRTI), which is associated with the development of dyslipidemia and dysregulation in glucose homeostasis [25,26,27,28,29].

The difference in metabolic syndrome prevalence between PLWH and the general population is likely due to the higher concentration of triglycerides and prevalence of diabetes and the lower HDL-c concentration observed in PLWH. The increased diabetes prevalence in PLWH observed in our study could possibly be associated with causes similar to the increased triglycerides concentration. Noubissi et al. [5] described an association between cART and diabetes, although Önen et al. [22] and Savès et al. [24] found no difference in diabetes prevalence between PLWH and the general population.

The PLWH cohort in our study was less often treated for hypertension, diabetes, and dyslipidemia than the Spanish general population. Prior studies in other countries have yielded contradictory results on this topic. Gelpi et al. [23] described a similar proportion of treated diabetes and hypertension between PLWH and the general population; on the other hand, the PLWH group had more dyslipidemia-treated patients than in the general population of Copenhagen. In Australia, Dharan et al. [30] also found that PLWH purchased more dyslipidemia medication but observed that fewer PLWH received treatment for diabetes compared with the non-infected population; the authors attributed these findings to differences in population characteristics between the two groups. In the United States, Önen et al. [22] estimated greater prescription of anti-hypertensive and lipid-lowering medications for PLWH than their general population counterparts. Among a number of possible reasons for undertreatment of CV risk factors in PLWH in Spain is the lower efficacy thresholds in PLWH of drugs used for treating metabolic disorders, particularly dyslipidemia [31]. Most of these drugs also have potential interactions with some antiretroviral drugs, particularly booted PI and non-nucleoside reverse transcriptase inhibitors (NNRTI); this may result in the use of suboptimal doses or slow titration that ultimately have an impact on treatment efficacy. All of these considerations support the need for a more thorough monitoring of CV risk factors in PLWH.

Lower HDL-c levels and lower prevalence of hypertension in PLWH were described in the French study by Savès et al. [24]; in contrast, we did not observe a lower prevalence of hypertension in our study. Up to three times less PLWH diagnosed with hypertension and/or diabetes were receiving treatment to manage these chronic conditions in our cohort compared to the general population. The treatment of dyslipidemia was also comparatively lower in PLWH, which can be explained by the lower concentrations of total cholesterol and LDL-c concentrations in PLWH than in the Spanish general population.

Many of these previous studies did not stratify PLWH characteristics by sex. Our study is one of the few to estimate CV risk factor prevalence separately in women and in men. Unlike men, WLWH had a higher glucose concentration and systolic blood pressure compared to women from the general population. These differences support the need for stratification by sex in future studies in order to better describe each gender’s characteristics and health necessities.

Our findings suggest that previous non-standardized CV risk-factor prevalence comparisons, such as those discussed above, of PLWH with the general population may have produced biased results due to the large difference of age in both populations. Our analysis pointed to this observation as we observed differences between crude and standardized estimates in both WLWH and MLWH. Standardization controls for the effect of age in different populations and allows for fair comparisons [24,32,33]. We used the direct method with European standard population weights. The results obtained after standardization and stratification by sex yield comparable values for both continuous and categorical factors.

## 5. Limitations

This study has three main limitations. First, its observational and cross-sectional design makes it strictly descriptive. Second, the treatment of dyslipidemia has been computed on the overall populations because the criteria for diagnosis did not match in the DARIOS and VACH cohorts. Third, in the VACH cohort, we have excluded a large number of patients because they were out of the age range or because they had missing values in CV risk factors. This fact can only influence our results against our hypothesis since patients with worse management were excluded.

## 6. Conclusions

After appropriate standardization to correct for the effect of age, triglyceride concentration and the prevalence of smoking and diabetes were higher in both sexes of PLWH compared to the general population. In addition, glucose concentration and systolic blood pressure were higher in WLWH than in women from the general population. Spanish PLWH were undertreated for diabetes, hypertension, and dyslipidemia compared to the general population. More thorough intervention to address these modifiable risk factors is needed and should become a part of routine HIV care.

## Figures and Tables

**Table 1:** Standardized and crude prevalence of cardiovascular risk factors in women from the general population and women living with HIV.

	Standardized Prevalence			Crude Prevalence		
Cardiovascular Risk Factor	General Population	People Living with HIV	*p*-Value	General Population	People Living with HIV	*p*-Value
	N = 15,159	N = 4495		N = 15,159	N = 4495	
Age, years				53.3 (53.2–53.5)	44.0 (43.8–44.2)	0.000
Age categories:						0.000
35–44, %				27.2 (26.5–27.9)	60.0 (58.5–61.4)	
45–54, %				26.6 (25.9–27.3)	33.1 (31.7–34.5)	
55–64, %				27.0 (26.3–27.7)	5.27 (4.64–5.97)	
65–74, %				19.2 (18.6–19.8)	1.69 (1.33–2.11)	
Systolic blood pressure, mmHg	122 (122–123)	125 (123–127)	0.016	124 (123–124)	120 (120–121)	<0.001
Diastolic blood pressure, mmHg	75 (75–76)	76 (75–77)	0.14	76 (75–76)	75 (74–75)	<0.001
Hypertension, %	27.4 (26.7–28.0)	28.6 (26.2–30.9)	0.336	29.8 (29.1–30.6)	18.1 (17.0–19.3)	<0.001
Treated hypertension, % ^a^	62.7 (60.7–64.7)	21.9 (18.4–25.4)	<0.001	74.8 (73.5–76.0)	19.5 (16.9–22.4)	<0.001
HDL-c, mg/dL	57 (57–58)	54 (53–56)	<0.001	57 (57–58)	53 (52–53)	<0.001
LDL-c, mg/dL	136 (135–137)	119 (114–123)	<0.001	137 (136–137)	112 (111–113)	<0.001
Triglycerides, mg/dL ^b^	99 (99–100)	134 (122–134)	<0.001	97.5 (97.5–98.5)	126 (125–129)	<0.001
Total cholesterol, mg/dL	214 (213–215)	202 (197–207)	<0.001	215 (214–216)	191 (190–193)	<0.001
Treated dyslipidaemia, % ^c^	14.0 (13.5–14.6)	9.0 (7.2–10.9)	<0.001	15.8 (15.2–16.4)	4.29 (3.72–4.93)	<0.001
Total cholesterol/HDL-c	3.9 (3.9–3.9)	4.0 (3.9–4.1)	0.193	3.9 (3.9–3.9)	3.9 (3.8–3.9)	0.704
Triglycerides/HDL-c ^b^	1.7 (1.7–1.8)	2.5 (2.5–2.7)	<0.001	1.7 (1.7–1.8)	2.5 (2.5–2.6)	<0.001
Glucose, mg/dL	97 (96–97)	100 (97–103)	0.015	97.4 (97.0–97.8)	94.6 (94.0–95.3)	<0.001
Diabetes, %	10.6 (10.1–11.1)	20.5 (18.3–22.7)	<0.001	11.6 (11.1–12.1)	15.3 (14.2–16.4)	<0.001
Treated diabetes, % ^d^	34.4 (31.5–37.2)	12.1 (8.50–15.7)	<0.001	45.0 (42.6–47.4)	8.15 (6.22–10.5)	<0.001
Body mass index, kg/m^2^	27.5 (27.4–27.7)	24.0 (23.5–24.6)	<0.001	27.7 (27.7–27.8)	23.6 (23.4–23.7)	0.000
Metabolic Syndrome, %	20.5 (19.8–21.1)	24.3 (21.4–27.2)	0.011	22.0 (21.3–22.7)	18.8 (17.3–20.3)	<0.001
Smoking, %	21.2 (20.6–21.9)	44.5 (42.5–46.6)	<0.001	19.5 (18.9–20.1)	54.7 (53.2–56.2)	0.000

Values are expressed as mean (95% confidence interval). ^a^ Among patients with history of hypertension. ^b^ Mean (95% confidence interval) were obtained with log-transformed values. ^c^ Among all cohort participants. ^d^ Among patients with history of diabetes. HDL-c, high-density lipoprotein cholesterol; LDL-c, low-density lipoprotein cholesterol.

**Table 2:** Standardized and crude prevalence of cardiovascular risk factors in men from the general population and men living with HIV.

	Standardized Prevalence			Crude Prevalence		
Cardiovascular Risk Factor	General Population	People Living with HIV	*p*-Value	General Population	People Living with HIV	*p*-Value
	N = 13,201	N = 14,738		N = 13,201	N = 14,738	
Age, years				53.8 (53.6–54.0)	45.2 (45.1–45.3)	0.000
Age categories:						0.000
35–44, %				26.3 (25.6–27.1)	52.9 (52.1–53.7)	
45–54, %				25.4 (24.7–26.2)	37.0 (36.2–37.7)	
55–64, %				27.4 (26.7–28.2)	7.66 (7.24–8.10)	
65–74, %				20.8 (20.1–21.5)	2.48 (2.24–2.75)	
Systolic blood pressure, mmHg	130 (129–130)	128 (127–129)	<0.001	131 (130–131)	125 (125–126)	<0.001
Diastolic blood pressure, mmHg	79 (79–80)	78 (77–78)	<0.001	79 (79–80)	77 (77–78)	<0.001
Hypertension, %	29.0 (28.2–29.7)	29.3 (28.1–30.4)	0.676	31.4 (30.6–32.2)	22.3 (21.6–23.0)	<0.001
Treated hypertension, % ^a^	59.3 (57.5–61.1)	26.5 (24.7–28.3)	<0.001	70.1 (68.7–71.5)	22.2 (20.8–23.6)	0.000
HDL-c, mg/dL	49 (48–49)	46 (45–47)	<0.001	48.6 (48.4–48.8)	44.9 (44.6–45.2)	<0.001
LDL-c, mg/dL	139 (138–140)	111 (109–113)	<0.001	139 (138–139)	106 (106–107)	0.000
Triglycerides, mg/dL ^b^	122 (121–122)	148 (148–149)	<0.001	122 (120–123)	147 (145–148)	<0.001
Total cholesterol, mg/dL	214 (213–216)	190 (188–192)	<0.001	214 (213–214)	182 (182–183)	0.000
Treated dyslipidaemia, % ^c^	15.1 (14.5–15.7)	9.9 (9.0–10.7)	<0.001	16.7 (16.0–17.4)	5.85 (5.48–6.24)	<0.001
Total cholesterol/HDL-c	4.6 (4.6–4.7)	4.5 (4.4–4.5)	<0.001	4.58 (4.56–4.61)	4.40 (4.37–4.43)	<0.001
Triglycerides/HDL-c ^b^	2.7 (2.5–2.7)	3.3 (3.3–3.7)	<0.001	2.6 (2.5–2.6)	3.4 (3.4–3.5)	<0.001
Glucose, mg/dL	104 (103–105)	103 (102–105)	0.324	105 (105–106)	99 (98–99)	<0.001
Diabetes, %	15.4 (14.8–16.0)	23.9 (22.8–24.9)	<0.001	16.9 (16.3–17.6)	18.3 (17.7–18.9)	0.003
Treated diabetes, % ^d^	40.0 (37.1–42.9)	13.5 (11.9–15.1)	<0.001	47.1 (44.9–49.3)	10.1 (9.01–11.3)	<0.001
Body mass index, kg/m^2^	28.1 (27.9–28.2)	24.4 (24.2–24.6)	<0.001	28.1 (28.1–28.2)	24.2 (24.1–24.2)	0.000
Metabolic Syndrome, %	27.9 (27.1–28.7)	30.4 (29.0–31.8)	0.002	28.7 (27.9–29.5)	26.1 (25.2–27.1)	<0.001
Smoking, %	33.6 (32.8–34.4)	51.3 (50.2–52.5)	<0.001	32.3 (31.5–33.1)	57.6 (56.8–58.4)	0.000

Values are expressed as mean (95% confidence interval). ^a^ Among patients with history of hypertension. ^b^ Mean (95% confidence interval) were obtained with log-transformed values. ^c^ Among all cohort participants. ^d^ Among patients with history of diabetes. HDL-c, high-density lipoprotein cholesterol; LDL-c, low-density lipoprotein cholesterol.

## Data Availability

The datasets generated during and/or analysed during the current study are available from the corresponding author on reasonable request.

## Supplementary Materials

The following are available online at https://www.mdpi.com/article/10.3390/jpm11111085/s1, Figure S1: Flow chart of the general population cohort (DARIOS) and the people living with HIV cohort (VACH).

## References

[1] Lee F.J., Amin J., Carr A. (2014). Efficacy of Initial Antiretroviral Therapy for HIV-1 Infection in Adults: A Systematic Review and Meta-Analysis of 114 Studies with up to 144 Weeks’ Follow-Up. PLoS ONE.

[2] Deeks S.G., Lewin S.R., Havlir D.V. (2013). The end of AIDS: HIV infection as a chronic disease. Lancet.

[3] d’Ettorre G., Ceccarelli G., Pavone P., Vittozzi P., de Girolamo G., Schietroma I., Serafino S., Giustini N., Vullo V. (2016). What happens to cardiovascular system behind the undetectable level of HIV viremia?. AIDS Res. Ther..

[4] Domingo P., Suarez-Lozano I., Gutierrez F., Estrada V., Knobel H., Palacios R., Antela A., Blanco J., Fulladosa X. (2019). Predictive factors of renal impairment in HIV-infected patients on antiretroviral therapy: Results from the VACH longitudinal cohort study. Nefrologia.

[5] Noubissi E.C., Katte J.C., Sobngwi E. (2018). Diabetes and HIV. Curr. Diabetes Rep..

[6] Schouten J., Wit F.W., Stolte I.G., Kootstra N.A., van der Valk M., Geerlings S.E., Prins M., Reiss P. (2014). Cross-sectional comparison of the prevalence of age-associated comorbidities and their risk factors between HIV-infected and uninfected individuals: The AGEhIV cohort study. Clin. Infect. Dis..

[7] Zanni M., Schouten J., Grinspoon S.K., Reiss P. (2014). Risk of coronary heart disease in patients with HIV infection. Nat. Rev. Cardiol..

[8] Estrada V., Bernardino J.I., Masiá M., Iribarren J.A., Ortega A., Lozano F., Miralles C., Olalla J., Santos J., Elias M.J.P. (2015). Cardiovascular risk factors and lifetime risk estimation in HIV-infected patients under antiretroviral treatment in Spain. HIV Clin. Trials.

[9] Masiá M., Pérez-Cachafeiro S., Leyes M., López-Aldeguer J., López M., Segura F., Blanco J.R., Peña A., Rodríguez F., Vera M. (2012). Cardiovascular risk in human immunodeficiency virus-infected patients in Spain. CoRIS cohort, 2011. Enferm. Infecc. Microbiol. Clin..

[10] Cuzin L., Katlama C., Cotte L., Pugliese P., Cheret A., Bernaud C., Rey D., Poizot-Martin I., Chirouze C., Bani-Sadr F. (2017). Ageing with HIV: Do comorbidities and polymedication drive treatment optimization?. HIV Med..

[11] Kaida A., Carter A., Nicholson V., Lemay J., O’Brien N., Greene S., Tharao W., Proulx-Boucher K., Gormley R. (2019). Hiring, training, and supporting Peer Research Associates: Operationalizing community-based research principles within epidemiological studies by, with, and for women living with HIV. Harm Reduct. J..

[12] Monforte A.D., Anderson J., Olczak A. (2013). What do we know about antiretroviral treatment of HIV in women. Antivir. Ther..

[13] UNAIDS-People Living with HIV-HIV Prevalence. https://aidsinfo.unaids.org/.

[14] Grau M., Elosua R., de León A.C., Guembe M.J., Baena-Díez J.M., Alonso T.V., Félix F.J., Zorrilla B., Rigo F., Lapetra J. (2011). Cardiovascular risk factors in Spain in the first decade of the 21st Century, a pooled analysis with individual data from 11 population-based studies: The DARIOS study. Rev. Española De Cardiol. Engl. Ed..

[15] Sistema DARIOS de Estimación de la Prevalencia de Combinaciones de Factores de Riesgo Cardiovascular en España en la Primera Década del Siglo XXI. http://darios.imim.es/.

[16] Suárez-Lozano I., Fajardo J.M., Garrido M., Roca B., García-Alcalde M.L., Geijo P., Selma D., Lozano F., Teira R., Viciana P. (2002). Epidemiological trends of HIV infection in Spain: Preventative plans have to be oriented to new target populations (Spanish VACH Cohort). Aids.

[17] Knobel H., Domingo P., Suarez-Lozano I., Gutierrez F., Estrada V., Palacios R., Antela A., Blanco J., Fulladosa X., Refollo E. (2019). Rate of cardiovascular, renal and bone disease and their major risks factors in HIV-infected individuals on antiretroviral therapy in Spain. Enferm. Infecc. Y Microbiol. Clin..

[18] WHO MONICA Project e-Publications. https://www.thl.fi/publications/monica/.

[19] ISD Services|Geography, Population and Deprivation Analytical Support Team|Population|Standard Populations|ISD Scotland. https://www.isdscotland.org/Products-and-Services/GPD-Support/Population/Standard-Populations/.

[20] Waterhouse J.A.H., Muir C.S., Correa P., Powell J. (1976). Cancer Incidence in Five Continents.

[21] R Core Team (2021). R: A Language and Environment for Statistical Computing.

[22] Önen N.F., Overton E.T., Seyfried W., Stumm E.R., Snell M., Mondy K., Tebas P. (2010). Aging and HIV infection: A comparison between older HIV-infected persons and the general population. HIV Clin. Trials.

[23] Gelpi M., Afzal S., Lundgren J., Ronit A., Roen A., Mocroft A., Gerstoft J., Lebech A.-M., Lindegaard B., Kofoed K. (2018). Higher Risk of Abdominal Obesity, Elevated Low-Density Lipoprotein Cholesterol, and Hypertriglyceridemia, but not of Hypertension, in People Living With Human Immunodeficiency Virus (HIV): Results From the Copenhagen Comorbidity in HIV Infection Study. Clin. Infect. Dis..

[24] Savès M., Chêne G., Ducimetière P., Leport C., Le Moal G., Amouyel P., Arveiler D., Ruidavets J.-B., Reynes J., Bingham A. (2003). Risk Factors for Coronary Heart Disease in Patients Treated for Human Immunodeficiency Virus Infection Compared with the General Population. Clin. Infect. Dis..

[25] da Cunha J., Maselli LM F., Stern AC B., Spada C., Bydlowski S.P. (2015). Impact of antiretroviral therapy on lipid metabolism of human immunodeficiency virus-infected patients: Old and new drugs. World J. Virol..

[26] (2019). EACS Guidelines 2019. http://www.eacsociety.org.

[27] Kanters S., Vitoria M., Doherty M., Socias M.E., Ford N., I Forrest J., Popoff E., Bansback N., Nsanzimana S., Thorlund K. (2016). Comparative efficacy and safety of first-line antiretroviral therapy for the treatment of HIV infection: A systematic review and network meta-analysis. Lancet HIV.

[28] Squires K., Kityo C., Hodder S., Johnson M., Voronin E., Hagins D., Avihingsanon A., Koenig E., Jiang S., White K. (2016). Integrase inhibitor versus protease inhibitor based regimen for HIV-1 infected women (WAVES): A randomised, controlled, double-blind, phase 3 study. Lancet HIV.

[29] Grupo de Estudio de Sida Documento de Consenso Sobre Alteraciones Metabólicas Y Riesgo Cardiovascular 3 En Pacientes Con Infección Por El VIH. https://www.mscbs.gob.es/ciudadanos/enfLesiones/enfTransmisibles/sida/publicaciones/profSanitarios/RecomendacionesMetabolicasGEAM_PNS_GESIDAAbril2017_FinalT.pdf.

[30] Dharan N.J., Radovich T., Che S., Petoumenos K., Juneja P., Law M., Huang R., McManus H., Polizzotto M.N., Guy R. (2020). Comorbidity Medications Are Dispensed to More People Receiving Antiretroviral Therapy for HIV Compared with the General Population in Australia. AIDS Res. Hum. Retrovir..

[31] Silverberg M.J., Leyden W., Hurley L., Go A.S., Jr C.P.Q., Klein D., Horberg M.A. (2009). Response to Newly Prescribed Lipid-Lowering Therapy in Patients With and Without HIV Infection. Ann. Intern. Med..

[32] Kakinami L., Block R.C., Adams M.J., Cohn S.E., Maliakkal B., Fisher S.G. (2013). Risk of cardiovascular disease in HIV, hepatitis C, or HIV/hepatitis C patients compared to the general population. Int. J. Clin. Pract..

[33] Durand M., Sheehy O., Baril J.G., Lelorier J., Tremblay C.L. (2011). Association between HIV infection, antiretroviral therapy, and risk of acute myocardial infarction: A cohort and nested case–control study using Quebec’s public health insurance database. JAIDS J. Acquir. Immune Defic. Syndr..

