# Supplementary material for: Standardized Comparison of Cardiovascular Risk Factors Prevalence in Spanish Women and Men Living with HIV and in the General Population
Source: J Pers Med. 2021 Oct 25;11(11):1085. doi: 10.3390/jpm11111085 (PMC8621654; doi:10.3390/jpm11111085)
Supplement: Supplementary file 1 [file jpm-11-01085-s001.zip › jpm-1390744-supplementary.pdf]

Supplementary Materials

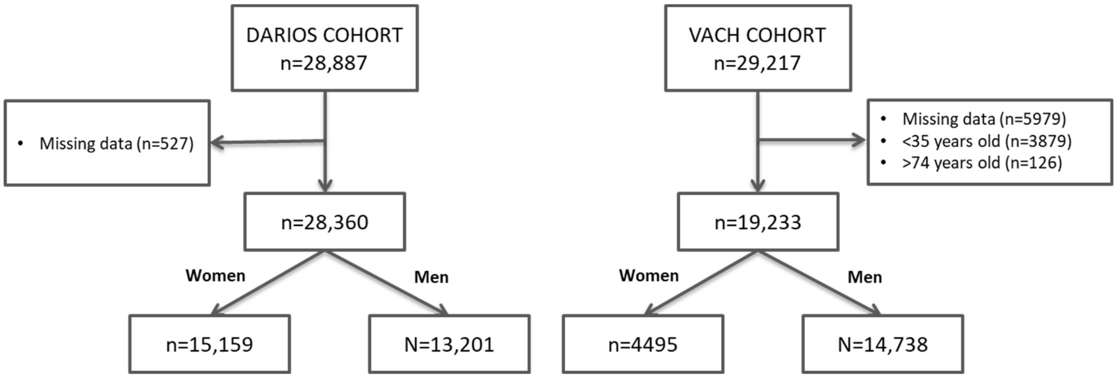

Figure S1. Flow chart of the general population cohort (DARIOS) and the people living with HIV cohort (VACH).
